# Author Correction: Proteomic characterization of gastric cancer response to chemotherapy and targeted therapy reveals potential therapeutic strategies

**DOI:** 10.1038/s41467-022-34238-0

**Published:** 2022-11-08

**Authors:** Yan Li, Chen Xu, Bing Wang, Fujiang Xu, Fahan Ma, Yuanyuan Qu, Dongxian Jiang, Kai Li, Jinwen Feng, Sha Tian, Xiaohui Wu, Yunzhi Wang, Yang Liu, Zhaoyu Qin, Yalan Liu, Jing Qin, Qi Song, Xiaolei Zhang, Akesu Sujie, Jie Huang, Tianshu Liu, Kuntang Shen, Jian-Yuan Zhao, Yingyong Hou, Chen Ding

**Affiliations:** 1 State Key Laboratory of Genetic Engineering and Collaborative Innovation Center for Genetics and Development, School of Life Sciences, Institute of Biomedical Sciences, Human Phenome Institute, Zhongshan Hospital, Fudan University, Shanghai, 200433 China; 2 Department of Pathology, Zhongshan Hospital, Fudan University, Shanghai, 200032 China; 3 State Key Laboratory of Cell Differentiation and Regulation, Henan International Joint Laboratory of Pulmonary Fibrosis, Henan center for outstanding overseas scientists of pulmonary fibrosis, College of Life Science, Institute of Biomedical Science, Henan Normal University, Xinxiang, 453007 China; 4 Department of Oncology, The Affiliated Hospital of Southwest Medical University, Luzhou, 646000 China; 5 Department of Urology, Fudan University Shanghai Cancer Center, Shanghai, 200032 China; 6 Department of Oncology, Shanghai Medical College, Shanghai, 200032 China; 7 Shanghai Genitourinary Cancer Institute, Shanghai, 200032 China; 8 Department of General Surgery, Zhongshan Hospital, Fudan University, Shanghai, 200032 China; 9 Department of Oncology, Zhongshan Hospital, Fudan University, Shanghai, 200032 China; 10 Institute for Developmental and Regenerative Cardiovascular Medicine, MOE-Shanghai Key Laboratory of Children’s Environmental Health, Xinhua Hospital, Shanghai Jiao Tong University School of Medicine, Shanghai, 200092 China; 11 Department of Anatomy and Neuroscience Research Institute, School of Basic Medical Sciences, Zhengzhou University, Zhengzhou, 450001 China

**Keywords:** Cancer therapeutic resistance, Gastric cancer, Gastric cancer, Gastric cancer

Correction to: *Nature Communications* 10.1038/s41467-022-33282-0, published online 29 September 2022

The original version of this Article contained an error in the title, which was previously incorrectly given as ‘: Proteomic characterization of gastric cancer response to chemotherapy and targeted therapy reveals new therapeutic strategies.’ The correct version states ‘potential’ in place of ‘new’. This has been corrected in both the PDF and HTML versions of the Article.

