# Supplementary material for: Proteomic characterization of gastric cancer response to chemotherapy and targeted therapy reveals potential therapeutic strategies
Source: Nat Commun. 2022 Sep 29;13:5723. doi: 10.1038/s41467-022-33282-0 (PMC9522856; doi:10.1038/s41467-022-33282-0)
Supplement: Supplementary file 1 — Supplementary Information [file 41467_2022_33282_MOESM1_ESM.pdf]

Supplementary Figure 1

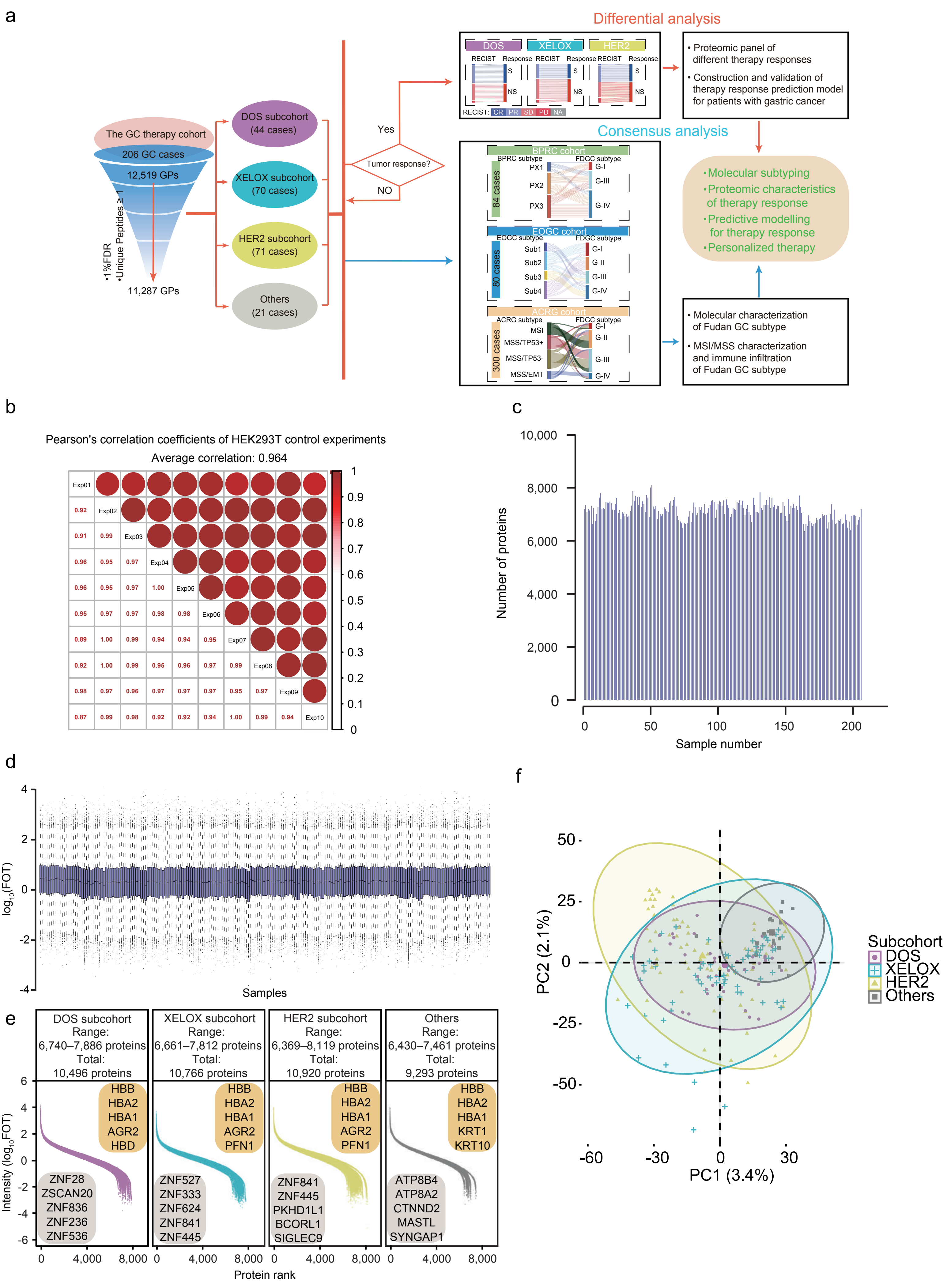

Supplementary Fig. 1. The workflow for proteomic analysis and quality control using the mass spectrometry platform. (a) Workflow showing data analysis, including consensus analysis and differential analysis. Consensus analysis were performed on the gastric cancer cohort, and the molecular subtyping was validated in BPRC, EOGC, and ACRG cohorts. The further differential expression analysis was performed on the DOS, XELOX, and HER2 subcohorts, and predictive models of therapy response for distinguishing the sensitive and non-sensitive groups were constructed. (b) Longitudinal quality control of MS using tryptic digests of HEK293T cells. The bottom-left half of the panel represents the pairwise Pearson's correlation coefficients of the samples (two-sided Pearson's correlation test), and the top-right half of the panel depicts the pairwise scatter plots from the same comparison. (c) Barplot for the identified protein number in the 206 GC cases. (d) Boxplot for log_{10} (FOT) in the 206 GC cases. Boxplots show median (central line), upper and lower quartiles (box limits), 1.5 × interquartile range (whiskers). (e) Overview of the proteomic profile of patients with GC. Shown are the dynamics of protein abundances identified in the DOS (purple), XELOX (blue), HER2 (yellow), and "Others" (gray) subcohorts. Proteins were quantified as a normalized intensity-based fraction of total (FOT) value and log_{10} transformed. The highest- and lowest-abundance proteins are shown in the box. (f) Principal components analysis (PCA) of the DOS (purple), XELOX (blue), HER2 (yellow), and "Others" (gray) subcohorts. Source data are provided as a Source Data file.

Supplementary Figure 2

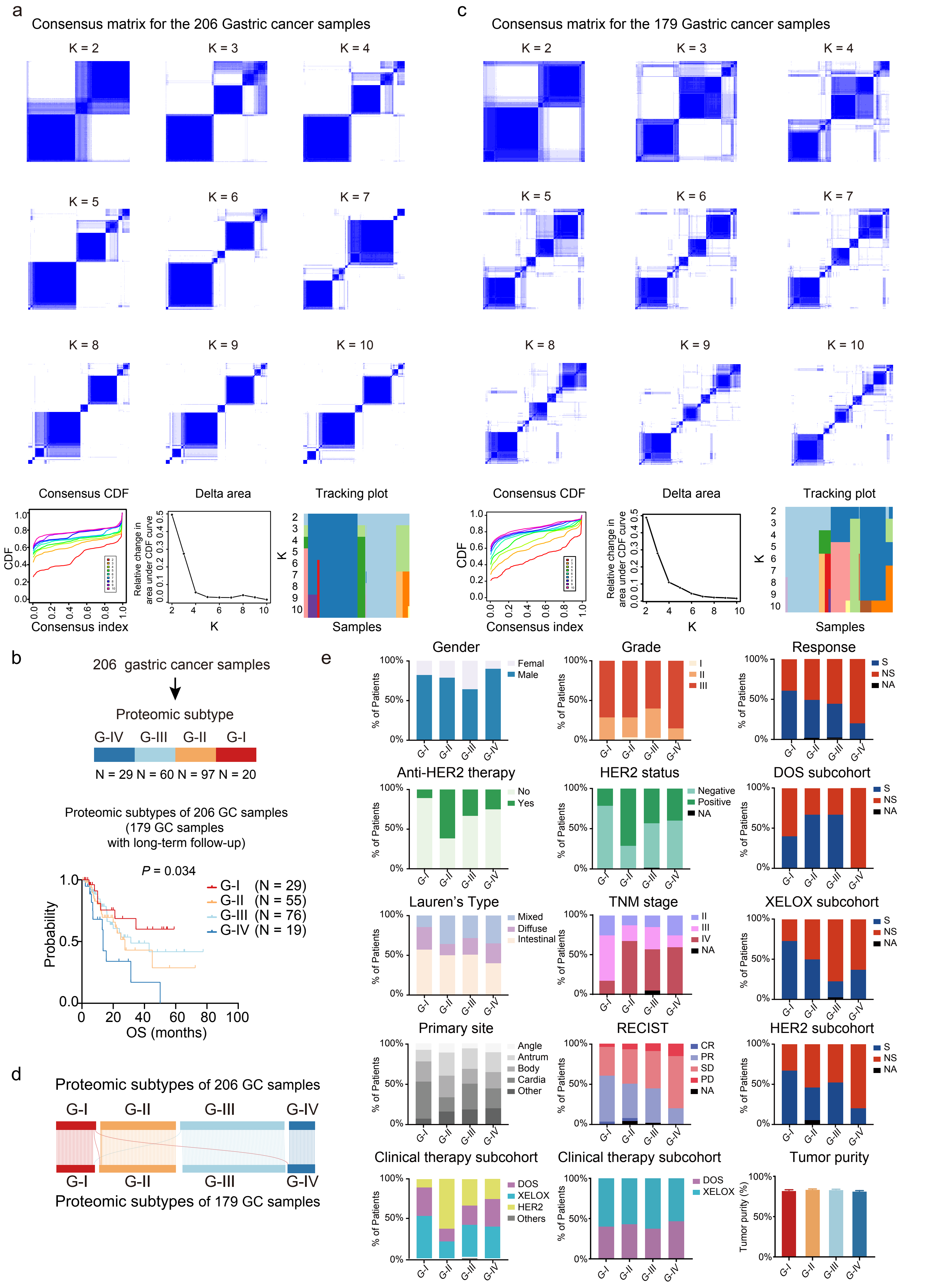

Supplementary Fig. 2. Consensus clustering analysis of the gastric cancer cohort identified four proteomic subtypes. (a) The consensus clustering analysis of 206 GC samples and four subtypes were generated. k was tested from 2 to 10. Consensus matrices, as well as the consensus cumulative distribution function (CDF) plot, delta area (change in CDF area) plot, and tracking plot are shown. (b) The Kaplan–Meier curves of overall survival (OS) of each proteomic subtype identified in 206 subtyping system (G-I, n = 29; G-II, n = 60; G-III, n = 97; and G-IV, n = 20). *P*-value is calculated by two-sided log rank test. (c) The consensus clustering analysis of 179 GC samples and four subtypes were generated. k is tested from 2 to 10. Consensus matrices, as well as the consensus cumulative distribution function (CDF) plot, delta area (change in CDF area) plot, and tracking plot are shown. (d) Sankey diagram indicating the comparison of proteomic subtypes between 206 GC samples and 179 GC samples. (e) The barplot for clinical characteristics in G-I–IV groups (such as gender, grade, Lauren's type, RECIST, and tumor purity, etc.).

Supplementary Figure 3

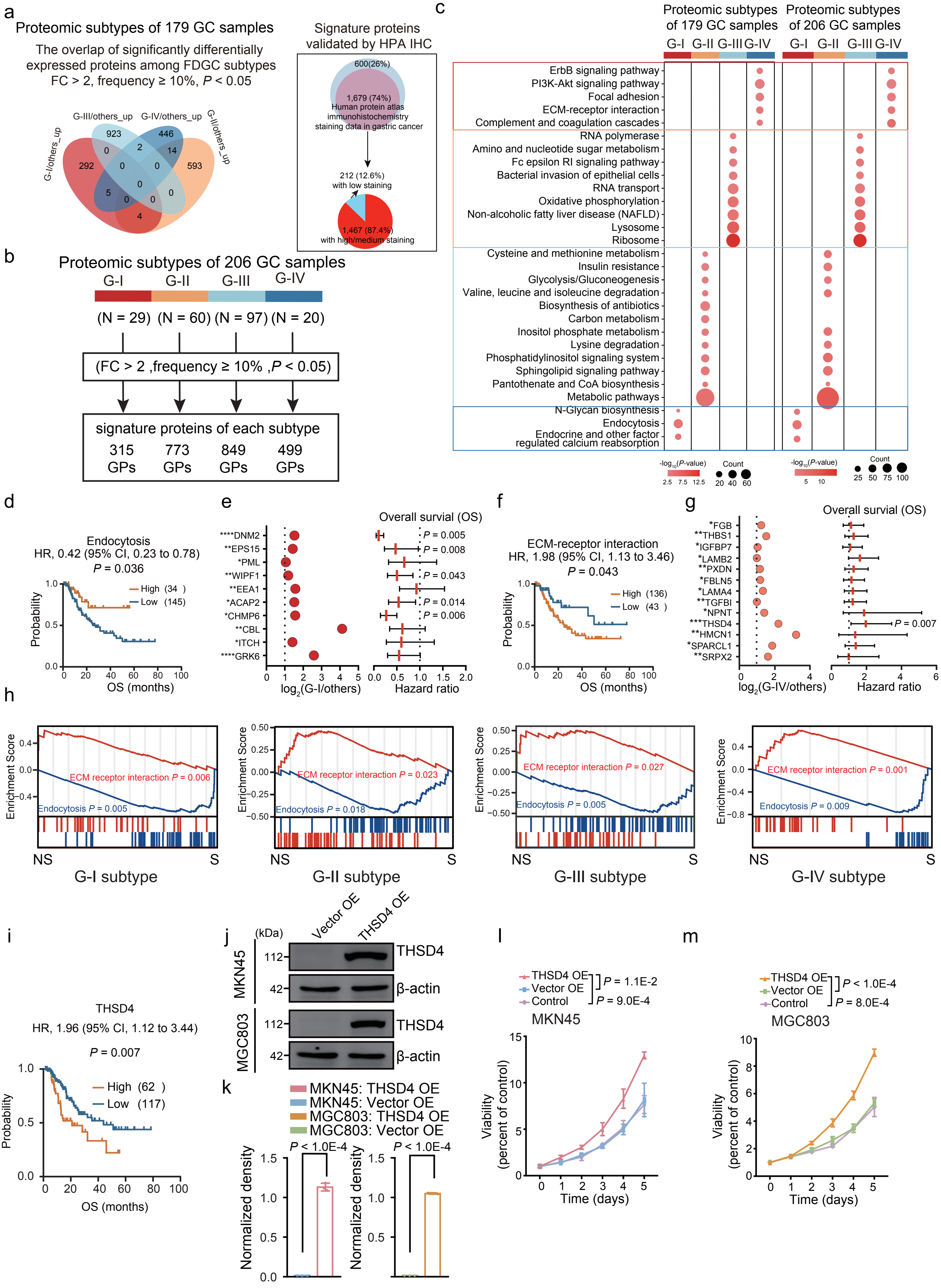

Supplementary Fig. 3. Proteomic subtypes and its molecular characteristics. (a, b) The overlap of significantly differentially expressed proteins (two-sided Student's t test, $P < 0.05$; fold change > 2) among proteomic subtypes of 179 GC samples (a) and 206 GC samples (b). These signature proteins of proteomic subtypes were mostly validated by Human Protein Atlas (HPA) Immunohistochemistry (IHC) Staining Data. (c) Bubble plot showing the KEGG pathway enrichment (two-sided Fisher's exact test) of four proteomic subtypes between 206 GC samples and 179 GC samples. (d, f) The association of endocytosis pathway (d) and ECM-receptor interaction pathway (f) score assessed by ssGSEA with OS (two-sided log rank test). (e, g) Left panel: The endocytosis related proteins upregulated in G-I subtype (e) and extracellular matrix (ECM) proteins upregulated in G-IV subtype (g) (two-sided Student's t test, $P < 0.05$; fold change > 2). Right panel: the red lines indicate the overall survival hazard ratios of endocytosis related proteins (e) and ECM proteins (g), and the endpoints represent lower or upper of the 95% confidence intervals. n = 179 biologically independent samples examined. The two-sided Cox P values are calculated. (h) GSEA enrichment analysis showing endocytosis pathway is enriched in S patients in four proteomic subtypes; while ECM pathway is enriched in NS patients in four proteomic subtypes (Nominal P value, calculated as Phenotype-based permutation test). (i) The Kaplan–Meier curves of the expression of ECM protein THSD4 with OS (two-sided log rank test). (j, k) Immunoblot analysis of THSD4 overexpression and the normalization of a qualified western blot. Data were analyzed by two-sided Student's t test and shown as mean ± SD (n = 3 independent experiments). (l, m) Effect of THSD4 overexpression on proliferation in gastric cancer (GC) cell lines (MKN45 and MGC803, n = 3 independent experiments, two-sided Student's t test, mean ± SD). Source data are provided as a Source Data file.

Supplementary Figure 4

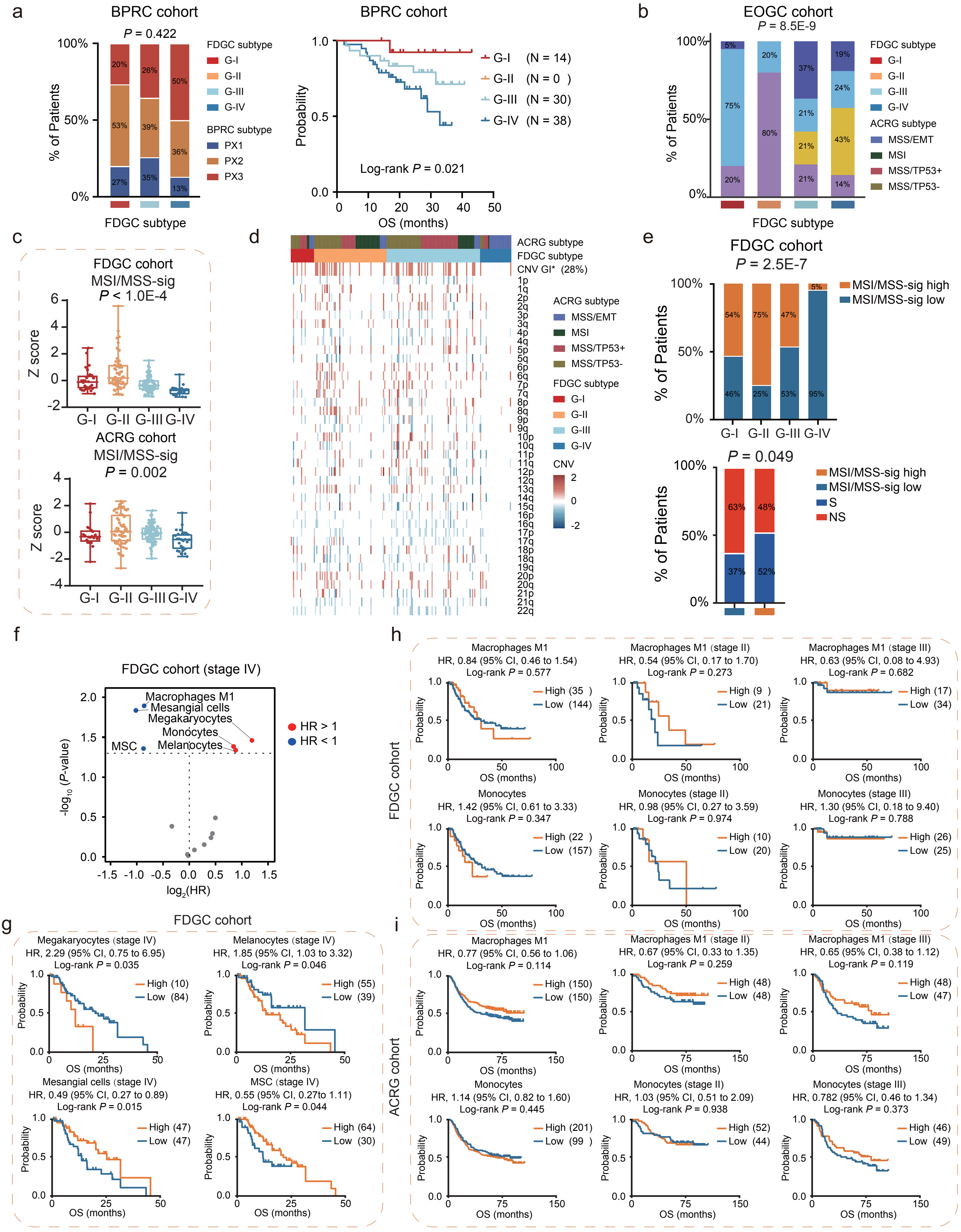

Supplementary Fig. 4. The validation of FDGC subtyping in other independent cohorts. (a) Barplot indicating the comparison of FDGC subtype and BPRC subtype. Survival analysis of BPRC cohort classified by FDGC subtyping (two-sided log rank test). (b) Barplot indicating the comparison of FDGC subtype and EOGC subtype (Chi-Squared Test). (c) Boxplot for MSI/MSS-sig evaluated by gene expression of signatures among four proteomic subtypes in FDGC cohort (n = 179 biologically independent samples) and ACRG cohort (n = 300 biologically independent samples) (two-way ANOVA test). Boxplots show median (central line), upper and lower quartiles (box limits), 1.5 × interquartile range (whiskers). (d) The copy number variations of ACRG cohort classified by FDGC subtyping. (e) Barplot for MSS/MSI-sig level among four subtypes, and the proportion of S and NS in MSI/MSS-sig high or low group (two-sided Fisher's exact test). (f) Volcano showing the association with OS of cell types with significant difference among four proteomic subtypes (two-sided log rank test). (g) The Kaplan–Meier curves of OS in FDGC cohort according the abundance of cell types (two-sided log rank test). (h, i) Survival analysis of monocytes and macrophages M1 in FDGC cohort (h) and ACRG cohort (i) according to different TNM stages (two-sided log rank test).

Supplementary Figure 5

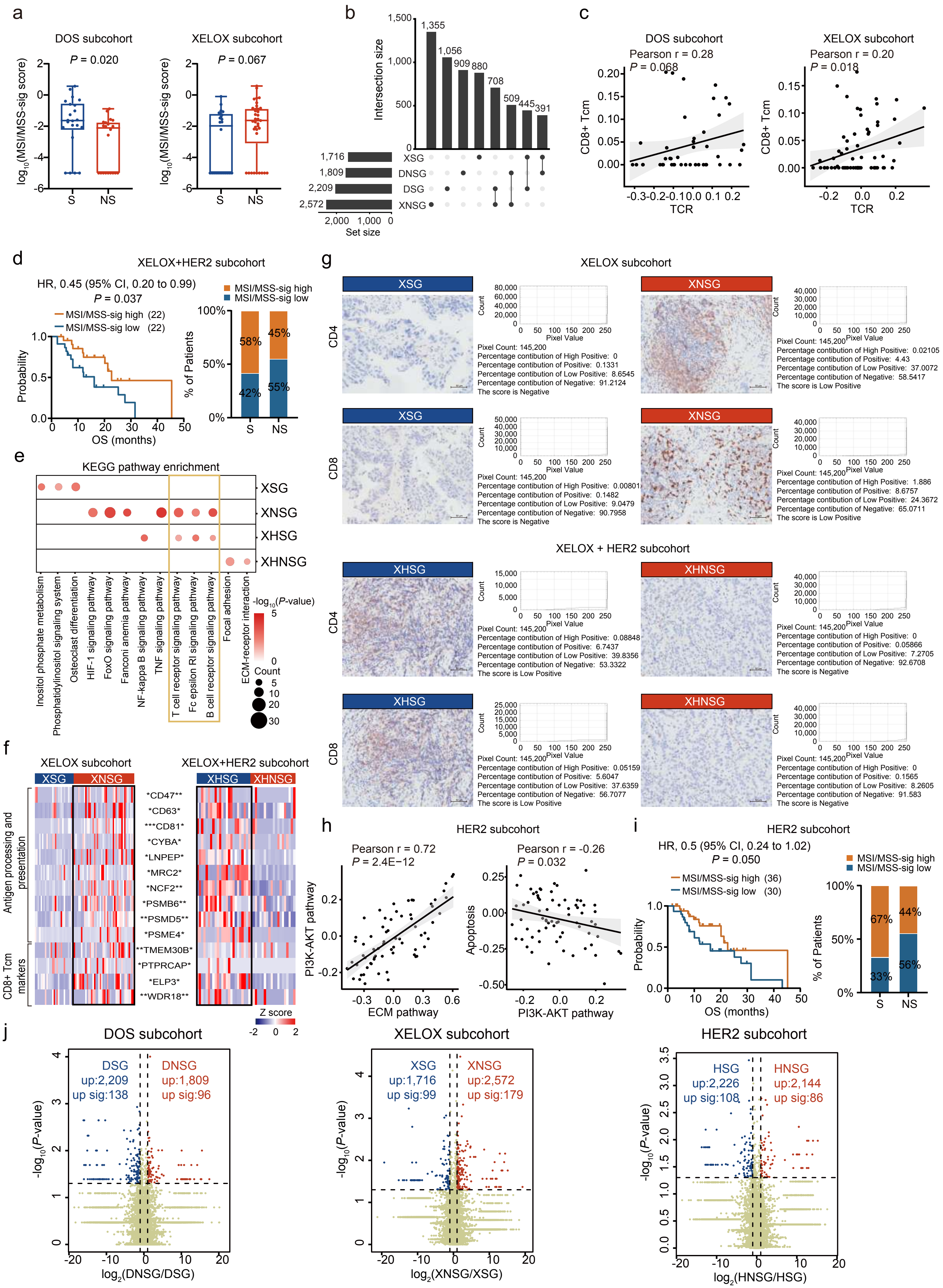

Supplementary Fig. 5. The differential analysis in the DOS, XELOX, and HER2 subcohorts. (a) Boxplot for MSS/MSI-sig level between S and NS in DOS [n (S) = 22, n (NS) = 22] and XELOX subcohorts [n (S) = 27, n (NS) = 42]. Boxplots show median (central line), upper and lower quartiles (box limits), 1.5 × interquartile range (whiskers). *P*-values are calculated by two-sided Student's t test. (b) The upset plot showing the identifications of overrepresented proteins in DSG, DNSG, XSG, and XNSG. (c) Correlation of CD8+ Tcm and TCR signaling pathway in DOS and XELOX subcohorts (two-sided Pearson's correlation test). (d) Barplot for MSS/MSI-sig characteristics between S and NS in XELOX + HER2 subcohort and its association with OS (two-sided log rank test). (e) Bubble plot showing the KEGG pathway enrichment (two-sided Fisher's exact test) of XSG, XNSG, XHSG, and XHNSG groups. (f) Heatmap illustrating the significantly differential expression of proteins related to antigen processing and presentation and CD8+ Tcm markers (two-sided Wilcoxon rank-sum test). (g) Immunohistochemistry (IHC) staining and qualification of CD4 and CD8 in representative examples in the XELOX and XELOX + HER2 subcohorts. n = 3 independent experiments performed. The representative results are shown. The scale bar indicates 50 μm. (h) Correlation of ECM pathway score and PI3K-AKT pathway score, PI3K-AKT pathway score and apoptosis pathway score (two-sided Pearson's correlation test). (i) Barplot for MSS/MSI-sig characteristics between S and NS in HER2 subcohort and its association with OS (two-sided log rank test). (j) Volcanos showing the differential expression of DOS, XELOX, and HER2 subcohorts (two-sided Wilcoxon rank-sum test). $^{*}P < 0.05$ is considered statistically significant. $^{*}P < 0.05$, $^{**}P < 0.01$, $^{***}P < 0.001$, $^{****}P < 0.0001$. Source data are provided as a Source Data file.

Supplementary Figure 6

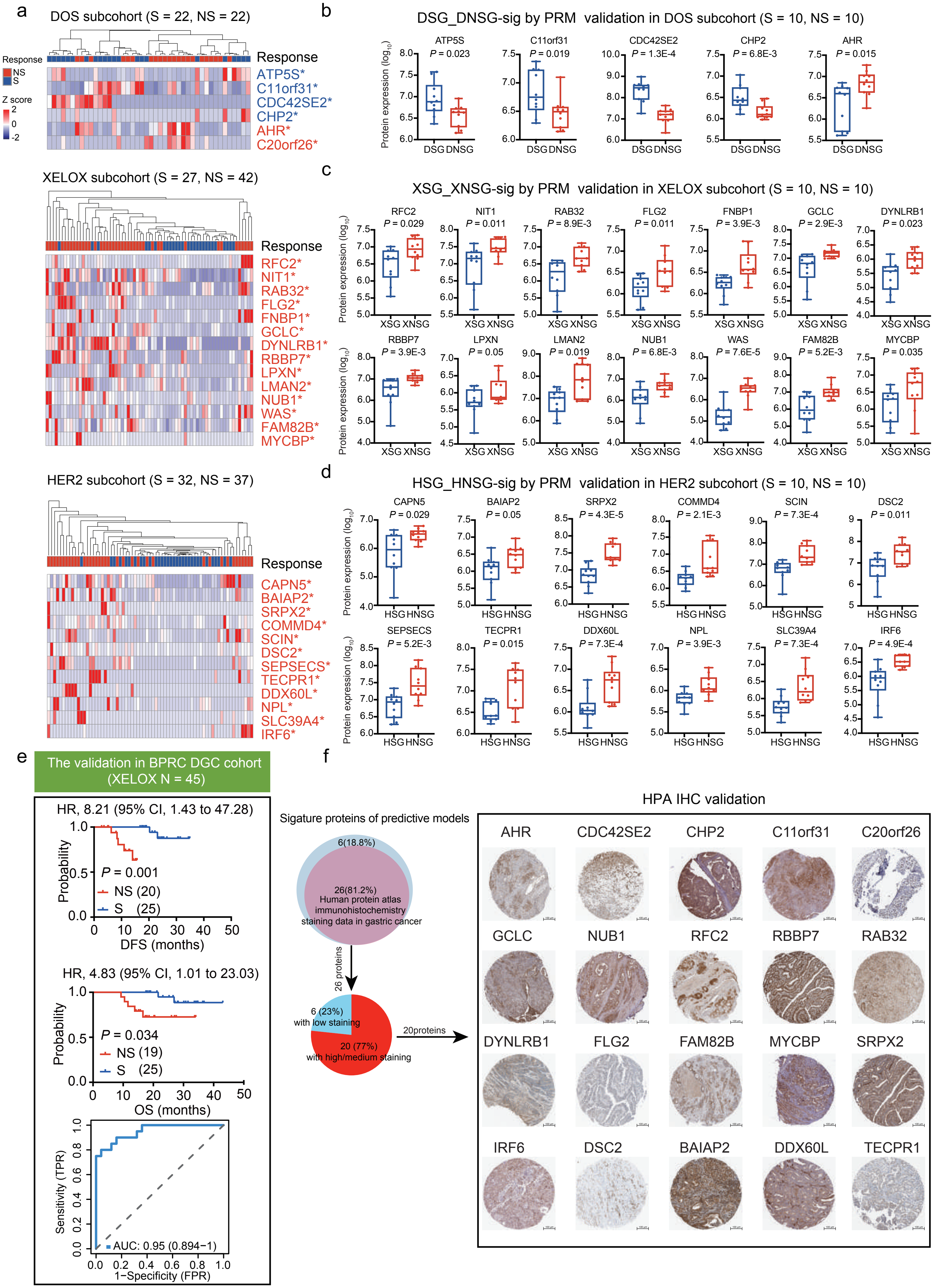

Supplementary Fig. 6. The signatures for predictive models in DOS, XELOX, and HER2 subcohorts and their validation in other cohorts. (a) Heatmaps of the DSG/DNSG-sig, XSG/XNSG-sig, HSG/HNSG-sig that discriminate between DSG and DNSG, XSG and XNSG, HSG and HNSG, respectively. *P*-values are calculated by two-sided Wilcoxon rank-sum test with Benjamini-Hochberg (BH) adjusted $P < 0.05$. (b–d) Boxplots showing the differential expression (Log10-transformed) of the DSG/DNSG-sig (b), XSG/XNSG-sig (c), and HSG/HNSG-sig (d) (two-sided Wilcoxon rank-sum test, n = 10 biologically independent samples in each group). Boxplots show median (central line), upper and lower quartiles (box limits), 1.5 × interquartile range (whiskers). (e) The validation cohort: the chemo-non-sensitive group [n (NS) = 20, median disease-free survival (mDFS) = 339.5 days] and the chemo-sensitive group [n (S) = 25, mDFS = 695.0 days] are defined. The Kaplan–Meier curves of DFS (n = 45) and OS (n = 44) of the S and NS groups (two-sided log rank test). The DFS and OS are calculated based on 30-day month. The ROC curves of XSG/XNSG-sig in predicting drug sensitivity in the validation cohort. (f) These signature proteins of predictive models are mostly validated by HPA IHC Staining Data. Representative IHC images for these signature proteins of predictive models. The independent experiments are performed at least 3 times. The representative results are shown. The scale bar indicates 100 μm. Source data are provided as a Source Data file.

Supplementary Figure 7

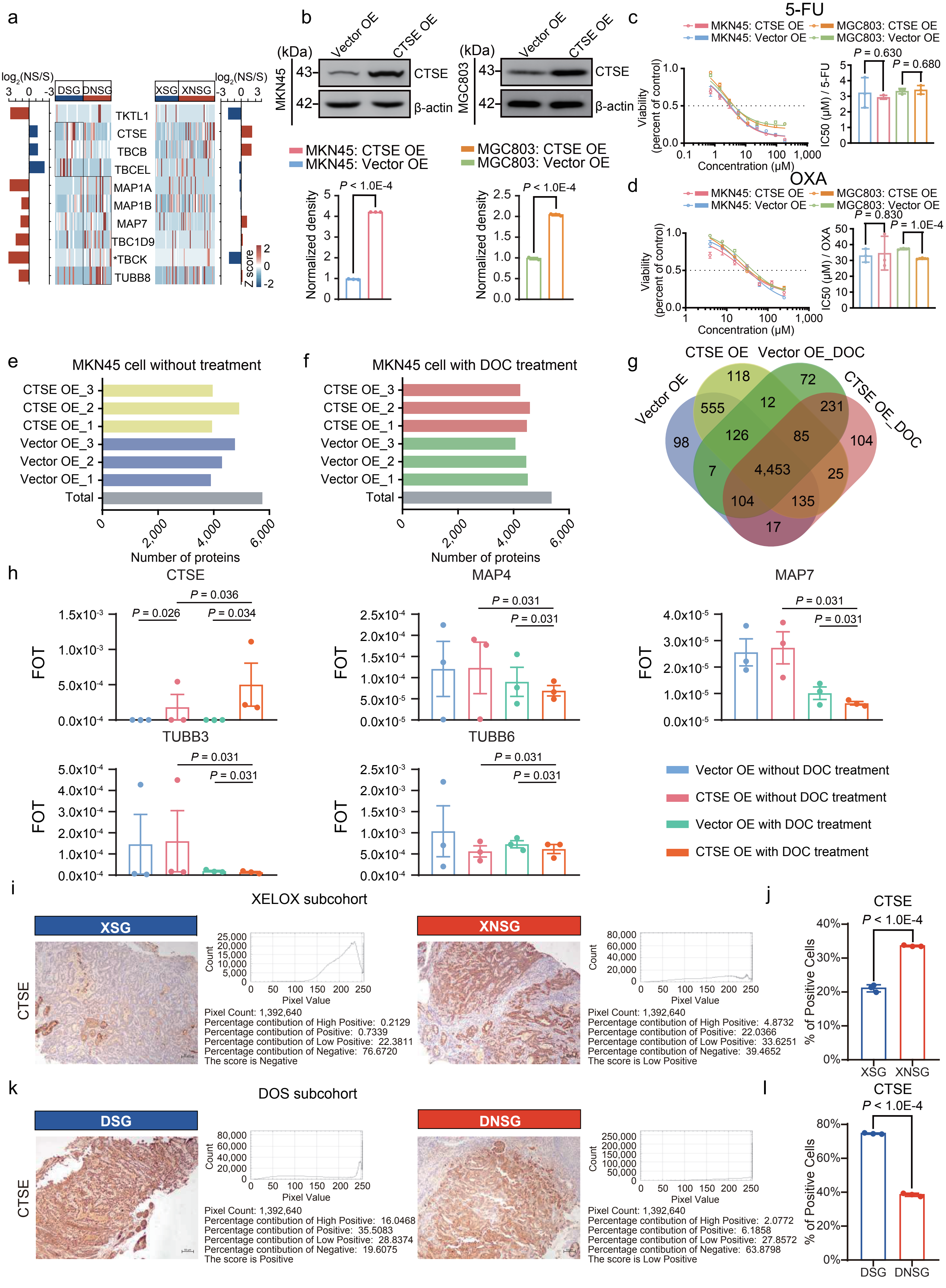

Supplementary Fig. 7. A possible mechanism by which CTSE modulates docetaxel sensitivity via microtubule stabilizing effects. (a) Heatmap of several key proteins in DSG/DNSG and XSG/XNSG. Barplot of fold change of these proteins. *P*-values are calculated by two-sided Wilcoxon rank-sum test. *$P < 0.05$ is considered statistically significant. (b) Immunoblot analysis of CTSE overexpression and the normalization of a qualified western blot (n = 3 independent experiments, two-sided Student's t test, mean ± SD). (c, d) Dose-response curves of GC cell lines (MKN45 and MGC803) overexpressing CTSE after 5-FU and OXA treatments, with an endpoint measurement at 72 h (n = 3 independent experiments, two-sided Student's t test, mean ± SD). (e, f) Barplot showing the numbers of identified proteins in MKN45 cells overexpressing CTSE with or without DOC treatment. (g) A Venn diagram showing the protein overlap of vector OE, CTSE OE, vector OE treated with DOC, and CTSE OE treated with DOC. (h) Effects of CTSE overexpression on the regulation of microtubule associated proteins in MKN45 cells after DOC treatment (n = 3 independent samples, two-sided Wilcoxon rank-sum test, mean ± SEM). (i–l) Immunohistochemistry (IHC) staining and qualification of CTSE in representative examples in the DOS and XELOX subcohorts. The scale bar indicates 50 μm. Data are analyzed by two-sided Student's t test and shown as mean ± SD (n = 3 independent experiments).

Supplementary Figure 8

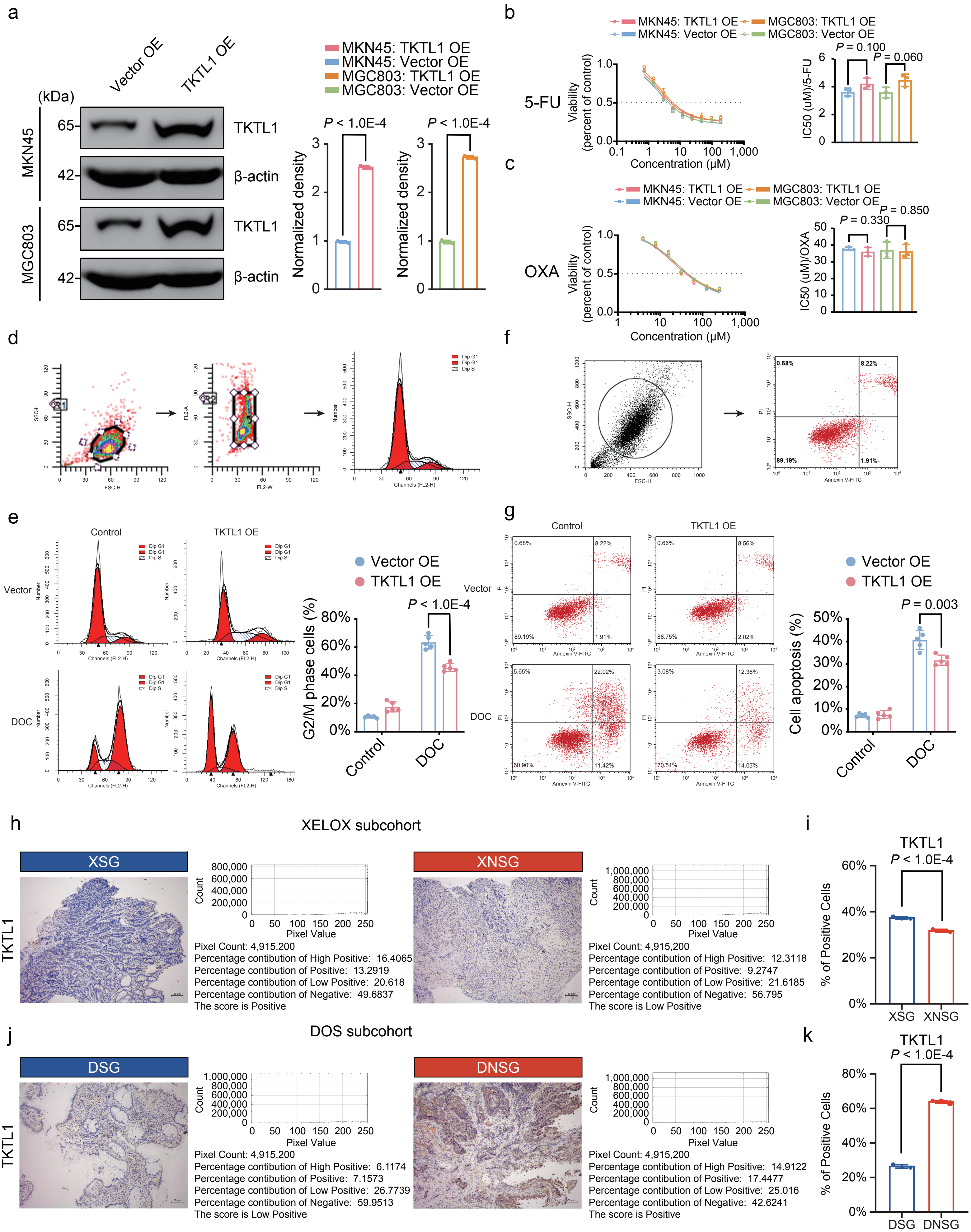

Supplementary Fig. 8. A possible mechanism by which TKTL1 modulates docetaxel sensitivity by inducing abnormal chromosome segregation. (a) Immunoblot analysis of TKTL1 overexpression and the normalization of a qualified western blot (n = 3 independent experiments, two-sided Student's t test, mean ± SD). (b, c) Dose-response curves of GC cell lines (MKN45 and MGC803) overexpressing TKTL1 after 5-FU and OXA treatments, with an endpoint measurement at 72 h (n = 3 independent experiments, two-sided Student's t test, mean ± SD). (d) Gating strategy for cell cycle FACS experiments. Cell population is gated in SSC-H/FSC-H to remove debris. "Cells" population is then gated in FL2-W/FL2-A to select only single cells. The "Singlets" population is represented as histogram over FL2-H. (e) Cell cycle was analyzed by PI staining and flow cytometry. Effects of TKTL1 overexpression and DOC treatment on the G2/M phase content of MKN45 cells (n = 3 independent experiments, two-sided Student's t test, mean ± SD). (f) Gating strategy for apoptosis FACS experiments. (g) Apoptotic cells were stained with Annexin V-FITC/PI and analyzed by flow cytometry. Cell apoptosis in MKN45 cells with various treatments (n = 3 independent experiments, two-sided Student's t test, mean ± SD). (h–k) Immunohistochemistry (IHC) staining and qualification of TKTL1 in representative examples in the DOS and XELOX subcohorts. The scale bar indicates 50 μm. Data are analyzed by two-sided Student's t test and shown as mean ± SD (n = 3 independent experiments). Source data are provided as a Source Data file.

Supplementary Table 1: Therapy regimens in GC cohort.

		DOS subcohort (n=44)	XELOX subcohort (n=70)	HER2 subcohort (n=71)	Others (n=21)	Whole GC cohort (n=206)
Docetaxel<br>Oxaliplatin<br>S-1		44 (100.00)	-	-	-	44 (21.36)
Oxaliplatin<br>Capecitabine		-	70 (100.00)	-	-	70 (33.98)
Trastuzumab		-	-	2 (2.82)	-	2 (0.97)
Trastuzumab plus	Docetaxel	-	-	3 (4.23)	-	3 (1.46)
	5-fluorouracil	-	-	6 (8.45)	-	6 (2.91)
	Camptothecin	-	-	3 (4.23)	-	3 (1.46)
	Platinum	-	-	1 (1.41)	-	1 (0.49)
	Pertuzumab	-	-	1 (1.41)	-	1 (0.49)
	Oxaliplatin<br>Capecitabine	-	-	46 (64.79)	-	46 (22.33)
	5-fluorouracil<br>Docetaxel	-	-	9 (12.68)	-	9 (4.37)
5-fluorouracil<br>Docetaxel		-	-	-	1 (4.76)	1 (0.49)
Apatinib<br>Oxaliplatin<br>Capecitabine		-	-	-	2 (9.52)	2 (0.97)
not available		-	-	-	18 (85.71)	18 (8.74)

Supplementary Table 2: Formal statistical tests-for-interaction analysis for the association of tumor classification with treatment responses.

Tumor classification	HR (95%CI)	*P*	HR (95%CI)	*P* for interaction
Subtype	1.20 (0.54 to 2.7)	0.66	1.42	0.43
Gender	0.42 (0.04 to 4.5)	0.47	(0.58 to 3.45)	
Subtype	0.50 (0.08 to 3.07)	0.45	1.02	0.20
Age	0.95 (0.87 to 1.03)	0.21	(0.99 to 1.05)	
Subtype	3.09 (0.37 to 31.63)	0.32	0.79	0.56
Grade	3.27 (0.44 to 29.76)	0.26	(0.35 to 1.7)	
Subtype	2.29 (1.01 to 5.39)	0.05	0.82	0.34
Lauren's type	1.56 (0.52 to 4.76)	0.43	(0.54 to 1.24)	
Subtype	1.44 (0.78 to 2.73)	0.25	1.06	0.69
Primary site	0.97 (0.43 to 2.17)	0.94	(0.79 to 1.45)	
Subtype	1.77 (1.15 to 2.81)	0.0112	0.76	0.44
HER2 status	1.72 (0.26 to 11.3)	0.57	(0.37 to 1.54)	
Subtype	1.64 (0.64 to 4.39)	0.31	0.98	0.95
Therapy subcohort	1.10 (0.32 to 3.78)	0.88	(0.62 to 1.57)	
Subtype	2.41 (0.59 to 10.89)	0.23	0.87	0.56
TNM stage	1.76 (0.53 to 6.26)	0.37	(0.56 to 1.36)	

HR, hazard ratio; CI, confidence interval

Supplementary Table 3: Univariate and multivariate analysis of overall survival of GC patients in proteomic subtypes.

Variable (N)	Univariate analysis		Multivariate analysis	
	HR (95% CI)	*P* value	HR (95% CI)	*P* value
Subtype				
G-I (N=28)	1			
G-II (N=56)	2.40 (0.90 to 6.60)	0.079	2.64 (0.91 to 7.69)	0.075
G-III (N=75)	2.10 (0.78 to 5.40)	0.145	1.88 (0.69 to 5.13)	0.214
G-IV (N=20)	5.00 (1.76 to 14.40)	0.003	3.84 (1.24 to 11.91)	0.02
P trend	1.60 (1.20 to 2.10)	0.002	1.49 (1.10 to 2.02)	0.009
Therapy subcohort				
DOS (N = 44)	1			
XELOX (N = 65)	1.00 (0.43 to 2.50)	0.931		
HER2 (N = 68)	2.00 (0.88 to 4.50)	0.1		
Response				
NS (N=97)	1			
S (N=79)	0.56 (0.33 to 0.94)	0.027	0.16 (0.05 to 0.53)	0.003
TNM stage				
N=175	1.80 (1.20 to 2.70)	0.006	1.64 (1.08 to 2.50)	0.021
RECIST (N=176)	2.30 (1.50 to 3.60)	<0.001	6.15 (2.77 to 13.66)	<0.001
Laurens's type				
Diffuse (N=37)	1			
Intestinal (N=90)	0.35 (0.19 to 0.67)	0.001	0.45 (0.22 to 0.92)	0.029
Mixed type (N=52)	0.50 (0.25 to 1.00)	0.048	0.54 (0.25 to 1.15)	0.112
Grade				
Grade (N=179)	1.80 (1.00 to 3.20)	0.037	1.54 (0.81 to 2.95)	0.19
Gender				
Female (N = 46)	1			
Male (N = 133)	1.00 (0.57 to 1.90)	0.895		
Age (N 179)	1.00 (0.97 to 1.00)	0.865		

HR, hazard ratio; CI, confidence interval

Supplementary Table 4: Univariate and multivariate analysis of disease-free survival of ERBB2 in XELOX + HER2 subcohort.

Variable (N)	Univariate analysis		Multivariate analysis	
	HR (95% CI)	*P* value	HR (95% CI)	*P* value
ERBB2(N=62)	1.3(1 to 1.6)	0.035	1.497(1.169 to 1.92)	0.001
Gender(N=62)				
Female(N=15)				
Male(N=47)	0.66(0.29 to 1.5)	0.318		
Age(N=62)	0.98(0.94 to 1)	0.333		
Lauren's type				
Diffuse(N=16)				
Intestinal(N=28)	0.16(0.061 to 0.43)	<0.001	0.269(0.0816 to 0.89)	0.031
Mixed type(N=18)	0.53(0.225 to 1.24)	0.141	0.764(0.273 to 2.14)	0.609
Grade(N=62)	8.2(1.1 to 58)	0.036	4.114(0.556 to 30.44)	0.166
RECIST				
PD(N=5)				
PR(N=26)	0.017(0.0029 to 0.095)	<0.001	0.021(0.0027 to 0.16)	<0.001
SD(N=31)	0.018(0.033 to 0.102)	<0.001	0.017(0.017 to 0.0023)	<0.001
Stage(N=62)	1.7（0.91 to 3.2）	0.096		

HR, hazard ratio; CI, confidence interval
