# Supplementary material for: Proteomic characterization of gastric cancer response to chemotherapy and targeted therapy reveals potential therapeutic strategies
Source: Nat Commun. 2022 Sep 29;13:5723. doi: 10.1038/s41467-022-33282-0 (PMC9522856; doi:10.1038/s41467-022-33282-0)
Supplement: Supplementary file 3 — Description of Additional Supplementary Files [file 41467_2022_33282_MOESM3_ESM.pdf]

**Description of Additional Supplementary Files**

File Names: Supplementary Data 1

Description: **Clinical characteristics of 206 GC patients. (A)** Clinical characteristics of 206 GC patients. **(B)** GC Patient demographics and baseline characteristics.

File Names: Supplementary Data 2

Description: **Proteomic matrix of the 206 GC patients. (A)** The protein expression matrix of 12,519 proteins after match between runs. **(B)** The protein expression at least 1 unique peptide with 1% FDR of all 206 samples.

File Names: Supplementary Data 3

Description: **Molecular subtypes of GC. (A)** The proteomic subtype of the 206 GC patients. **(B)** The proteomic subtype of the 179 GC patients. **(C)** The association of the proteomic subtypes of 206 GC patients and 179 GC patients. **(D)** The significantly up-regulated expressed proteins in the proteomic subtypes of the gastric cancer cohort. **(E)** The pathway alterations in the proteomic subtypes of the gastric cancer cohort enriched by these signatures of these subtypes. (**F**) The clinical groups in an independent validation subcohorts. (**G**) Targeted peptides from ECM proteins. Peptides were identified from the library search result, and targeted in a PRM experiment. (**H**) The ECM proteins' expression by PRM assay in the DOS validation subcohort.

File Names: Supplementary Data 4

Description: **Application of FDGC subtype. (A)** The FDGC subtyping model was applied to the BPRC cohort. **(B)** The FDGC subtyping model was applied to the EOGC cohort. **(C)** The FDGC subtyping model was applied to the ACRG cohort.

File Names: Supplementary Data 5

Description: **The xCell analysis result of GC cohort.** The xCell analysis result of FDGC cohort **(A)**, ACRG cohort **(B)**, BPRC cohort **(C). (D)** Differential cell types among four subtypes of 179 proteomic subtyping**. (E)** The significantly down-regualted immune-related proteins in G-IV

subtype.

File Names: Supplementary Data 6

Description: **The differential analysis of DOS subcohort. (A)** The group of DOS subcohort. **(B)** The differentially expressed proteins of DSG and DNSG. **(C)** The pathway alterations of DSG and DNSG.

File Names: Supplementary Data 7

Description: **The differential analysis of XELOX subcohort. (A)** The group of XELOX subcohort. **(B)** The differentially expressed proteins of XSG and XNSG. **(C)** The pathway alterations of XSG and XNSG.

File Names: Supplementary Data 8

Description: **The differential analysis of XELOX combined with HER2 subcohort. (A)** The group of XELOX combined with HER2 subcohort. **(B)** The differentially expressed proteins of XHSG and XHNSG. **(C)** The pathway alterations of XHSG and XHNSG.

File Names: Supplementary Data 9

Description: **The differential analysis of HER2 subcohort. (A)** The group of HER2 subcohort. **(B)** The differentially expressed proteins of HSG and HNSG. **(C)** The pathway alterations of HSG and HNSG.

File Names: Supplementary Data 10

Description: **A list of signatures of the predictive classifiers in therapy subcohorts.** A list of signatures of the predictive classifiers in DOS subcohort **(A)**, XELOX subcohort **(B)**, HER2 subcohort **(C)**.

File Names: Supplementary Data 11

Description: **The PRM validation of the predictive signatures in an independent validation subcohorts. (A)** The clinical groups in an independent validation subcohorts. (**B**) Targeted

peptides from predictive signatures. Peptides were identified from the library search result, and targeted in a PRM experiment. **(C)** The signature proteins' expression by PRM assay in the DOS validation subcohort. **(D)** The signature proteins' expression by PRM assay in the XELOX validation subcohort. **(E)** The signature proteins' expression by PRM assay in the HER2 validation subcohort.

File Names: Supplementary Data 12

Description: **The differentially expressed proteins and pathway alterations in MKN45 cells overexpressing CTSE. (A)** The up-regulated proteins and down-regulated proteins of CTSE OE group. **(B)** KEGG pathways were obtained from enrichment analysis of differentially expressed proteins.

File Names: Supplementary Data 13

Description: **The differentially expressed proteins and pathway alterations in MKN45 cells overexpressing CTSE after DOC treatment. (A)** The up-regulated proteins and down-regulated proteins of CTSE OE group after DOC treatment. **(B)** KEGG pathways were obtained from enrichment analysis of differentially expressed proteins.
