# Supplementary material for: Proteomic characterization of gastric cancer response to chemotherapy and targeted therapy reveals potential therapeutic strategies
Source: Nat Commun. 2022 Sep 29;13:5723. doi: 10.1038/s41467-022-33282-0 (PMC9522856; doi:10.1038/s41467-022-33282-0)
Supplement: Supplementary file 17 — Reporting Summary [file 41467_2022_33282_MOESM17_ESM.pdf]

nature portfolio

Corresponding author(s): Chen Ding

Last updated by author(s): Aug 22, 2022

Reporting Summary

Nature Portfolio wishes to improve the reproducibility of the work that we publish. This form provides structure for consistency and transparency in reporting. For further information on Nature Portfolio policies, see our Editorial Policies and the Editorial Policy Checklist.

Statistics

For all statistical analyses, confirm that the following items are present in the figure legend, table legend, main text, or Methods section.

n/a	Confirmed	
	☒	The exact sample size (*n*) for each experimental group/condition, given as a discrete number and unit of measurement
	☒	A statement on whether measurements were taken from distinct samples or whether the same sample was measured repeatedly
	☒	The statistical test(s) used AND whether they are one- or two-sided<br>*Only common tests should be described solely by name; describe more complex techniques in the Methods section.*
	☒	A description of all covariates tested
	☒	A description of any assumptions or corrections, such as tests of normality and adjustment for multiple comparisons
	☒	A full description of the statistical parameters including central tendency (e.g. means) or other basic estimates (e.g. regression coefficient) AND variation (e.g. standard deviation) or associated estimates of uncertainty (e.g. confidence intervals)
	☒	For null hypothesis testing, the test statistic (e.g. *F*, *t*, *r*) with confidence intervals, effect sizes, degrees of freedom and *P* value noted<br>*Give P values as exact values whenever suitable.*
☒		For Bayesian analysis, information on the choice of priors and Markov chain Monte Carlo settings
	☒	For hierarchical and complex designs, identification of the appropriate level for tests and full reporting of outcomes
	☒	Estimates of effect sizes (e.g. Cohen's *d*, Pearson's *r*), indicating how they were calculated

Our web collection on statistics for biologists contains articles on many of the points above.

Software and code

Policy information about availability of computer code

Data collection	Samples were analysed on a Q Exactive HF-X mass spectrometer (Thermo Fisher Scientific, Rockford, IL, USA) coupled with high-performance liquid chromatography (EASY-nLC 1200 System, Thermo Fisher Scientific). The mass spectrometry data were acquired using the Xcalibur software v2.2 (Thermo Fischer Scientific).
Data analysis	Database searching for the mass spectrometry raw data were performed using Firmiana proteomics workstation, MaxQuant (1.6.2.10) software, and Skyline-daily (4.2.1.19004, University of Washington, USA); Statistical analyses were realized by R (v3.5.1). Approaches or algorithms used for the proteome data annotation include ConsensusClusterPlus (version 3.8), GSVA R package (v1.34.0), clusterProfiler R package (v3.18.1), pROC R package (version 1.16.2), Caret R package (version 6.0-86). Gene annotation was performed using online tools DAVID 6.8 and ConsensusPathDB. Results from the Fast Large Margin classifier model were built and validated using RapidMiner 9.6.0 (RapidMiner Inc, Boston, USA). Results from the western blot quantification was performed using ImageJ software (Version 1.52a, National Institutes of Health, MD, USA). Results from flow cytometric analysis were acquired using a Beckman Coulter flow cytometer (Beckman Coulter, Brea, CA, USA) and analyzed using FlowJo version 10.7.1 (Becton Dickinson Life Sciences). Standard statistical tests were used to analyze the clinical data, including but not limited to Student's t test, Wilcoxon rank-sum test, Fisher's exact test, Kruskal-Wallis test, Phenotype-based permutation test, ANOVA test Pearson's correlation test, log-rank test, Gehan-Breslow-Wilcoxon. Unless otherwise specified, all statistical tests were two-sided. To account for multiple-testing, the p values were adjusted using the Benjamini–Hochberg FDR correction. Kaplan–Meier plots (log-rank test, Gehan-Breslow-Wilcoxon) were used to describe survival analysis. Variables associated with overall survival were identified using univariate Cox proportional hazards regression models. Significant factors in univariate analysis were further subjected to a multivariate Cox regression analysis. All the analyses of clinical data were performed in R and GraphPad Prism. For functional experiments, each was repeated at least three times independently, and results were expressed as mean ± SEM or mean ± SD. Statistical analysis was performed using GraphPad Prism.

For manuscripts utilizing custom algorithms or software that are central to the research but not yet described in published literature, software must be made available to editors and reviewers. We strongly encourage code deposition in a community repository (e.g. GitHub). See the Nature Portfolio guidelines for submitting code & software for further information.

Data

Policy information about availability of data

All manuscripts must include a data availability statement. This statement should provide the following information, where applicable:

- Accession codes, unique identifiers, or web links for publicly available datasets
- A description of any restrictions on data availability
- For clinical datasets or third party data, please ensure that the statement adheres to our policy

The raw mass spectrometry (MS) proteomics data and parallel reaction monitoring (PRM)-MS proteomics data generated in this study have been deposited in the ProteomeXchange Consortium [dataset identifier: PXD024255 (http://proteomecentral.proteomexchange.org/cgi/GetDataset?ID=PXD024255)] via the iProX partner repository (http://www.iprox.cn/) under Project ID IPX0002116000 (https://www.iprox.cn/page/project.html?id=IPX0002116000). All the H&E-stained slides of tumor tissues in this study were deposited in Mendeley (https://data.mendeley.com/datasets/cv6ytf2fz7/1). The gene expression profiles of gastric cancer cell lines associated with drug sensitivity could be accessed at DepMap data portal (https://depmap.org/portal/). The gene expression profiles of gastric cancer cell lines in public dataset Expression 21Q2 in this study are available in the Depmap database (https://depmap.org/portal/download/?releasename=DepMap+Public+21Q2&filename=CCLE_expression.csv). HPA IHC Staining Data could be accessed at https://www.proteinatlas.org/. NCBI human Refseq protein database chould be accessed at https://www.ncbi.nlm.nih.gov/refseq/. Source data are provided with this paper. The remaining data are available within the Article, Supplementary Information or Source Data file.

Field-specific reporting

Please select the one below that is the best fit for your research. If you are not sure, read the appropriate sections before making your selection.

☒ Life sciences ☐ Behavioural & social sciences ☐ Ecological, evolutionary & environmental sciences

For a reference copy of the document with all sections, see nature.com/documents/nr-reporting-summary-flat.pdf

Life sciences study design

All studies must disclose on these points even when the disclosure is negative.

Sample size	To obtain the proteomic characterization of the gastric cancer response to chemo- and targeted therapies, we performed strict screening process on gastric cancer patients based on the standard treatments including XELOX therapy, DOS therapy, and anti-HER2-based therapy. At first, we screened 2,943 gastric cancer patients with therapies or surgeries from January 2002 to Match 2018 in the Department of Pathology of Zhongshan Hospital, Fudan University (Shanghai, R. P. China). Then, we excluded 2,737 patients with postoperative chemotherapy, chemoradiotherapy, none complete pathological information, and other factors. All the chemotherapy regimens were given at standard dosing according to guidelines for gastric cancer patients on National Comprehensive Cancer Network (https://www.nccn.org/). Briefly, the XELOX regimen was administered as follows: capecitabine (1000 mg/m2, twice daily on days 1–14) and oxaliplatin (130 mg/m2 on day 1) (PMID: 17522863). The DOS regimen was provided as S-1 (tegafur, gimeracil and oteracil porassium capsules; 40 mg/m2 orally administered twice a day on days 1–14), oxaliplatin (100 mg/m2 on day 1), and docetaxel (40 mg/m2 on day 1). The two regimens were repeated every 3 weeks (PMID: 29954358). The HER2-positive gastric cancer patients generally received the anti-HER2-based therapy either as a XELOX combined anti-HER2 therapy or other chemotherapies combined anti-HER2 therapy. The optimal regimen of trastuzumab in combination with oxaliplatin/ capecitabine in first-line treatment of HER2-positive advanced gastric cancer was administered as follows. Trastuzumab was administered at a loading dose of 8 mg/kg followed by 6 mg/kg infusion every 3 weeks (q3w); oxaliplatin was administered as 130 mg/m2 infusion, q3w, for up to 6 cycles; capecitabine (1000 mg/m2) was given orally twice daily on days 1–14, followed by a 7-day rest interval (PMID: 26857702). Lastly, we assembled two cohorts, of which archival FFPE tissues with at least 80% tumor purity of GC cases were taken from chemotherapy naive patients. The discovery cohort for proteome profiling consisted of 206 patients with GC, including the DOS subcohort (44 cases treated with S-1 and oxaliplatin combined with docetaxel), the XELOX subcohort (70 cases treated with capecitabine and oxaliplatin), and the HER2 subcohort (71 cases treated with the anti-HER2-based therapy), according to the treatment profiles. Another 21 cases were assigned as “Others,” of which 3 cases received apatinib or docetaxel therapies, and 18 cases had no chemotherapy information. The independent validation cohort for PRM verification included 60 patients with GC, receiving either DOS (N = 20), XELOX (N = 20), or anti-HER2 (N = 20) therapies. Written informed consent was received from all patients included in this study.
Data exclusions	No data were excluded from the analyses.
Replication	All experiments were reliably reproduced and results are represented as mean ± SEM or mean ± SD as appropriate, which is indicated in figure legends. The replicated analysis of 293T cell lysates were used for the QC of the mass spectrometer.
Randomization	The GC samples for proteome processing were randomized, as investigators were blinded to clinical information.
Blinding	For sample processing, PCA, consensus clustering analysis, multiple logistic regression analysis, all investigators were blinded to clinical information (including age, gender, grade, Lauren’s type, primary site).

Reporting for specific materials, systems and methods

We require information from authors about some types of materials, experimental systems and methods used in many studies. Here, indicate whether each material, system or method listed is relevant to your study. If you are not sure if a list item applies to your research, read the appropriate section before selecting a response.

Materials & experimental systems

n/a	Involved in the study
	☒ Antibodies
	☒ Eukaryotic cell lines
☒	Palaeontology and archaeology
☒	Animals and other organisms
	☒ Human research participants
☒	Clinical data
☒	Dual use research of concern

Methods

n/a	Involved in the study
☒	ChIP-seq
	☒ Flow cytometry
☒	MRI-based neuroimaging

Antibodies

Antibodies used

the mouse monoclonal anti-β-actin antibody (1:10,000 for Western blotting, Genscript, catalog No: A00702), the rabbit polyclonal anti-THSD4 antibody (1:2,000 for Western blotting, ABclonal, catalog No: A17773), the rabbit polyclonal anti-CTSE antibody (1:2,000 for Western blotting and 1:100 for Immunohistochemistry, Signalway Antibody, catalog No: 35666), the rabbit polyclonal anti-TKTL1 antibody (1:1,000 for Western blotting and 1:500 for Immunohistochemistry, Novus Biologicals, catalog No: NBP1-31674), the rabbit polyclonal anti-alpha-tubulin antibody (1:2,000 for Western blotting, Proteintech, catalog No: 11224-1-AP), and the rabbit polyclonal anti-beta-tubulin antibody (1:2,000 for Western blotting, Proteintech, catalog No: 10094-1-AP), the rabbit antibody monoclonal against CD4 (1:250 for Immunohistochemistry, GeneTech, catalog No: GT219107), the mouse monoclonal antibody against CD8 (1:300 for Immunohistochemistry, Leica, catalog NO: PA0183), the rabbit monoclonal anti-HER-2/NEU (4B5) antibody (working solution with the concentration of 6 μg/mL for Immunohistochemistry, Ventana Medical Systems, Inc. Tucson, AZ, USA).

Validation

1. Anti-β-actin antibody: Application statement in manufacturer's website as following: this anti-β-actin antibody is validated for use in WB and ELISA. https://www.genscript.com/antibody/A00702-THE_beta_Actin_Antibody_mAb_Mouse.html?position_no=1&sensors=search%20product%20box
2. Anti-THSD4 antibody: Application statement in manufacturer's website as following: this anti-THSD4 antibody is validated for use in WB. https://abclonal.com.cn/catalog/A17773
3. Anti-CTSE antibody: Application statement in manufacturer's website as following: this anti-CTSE antibody is validated for use in WB and IHC. https://www.sabbiotech.com.cn/g-8917-CTSE-Antibody-35666.html
4. Anti-TKTL1 antibody: Application statement in manufacturer's website as following: this anti-TKTL1 antibody is validated for use in WB, ICC/IF, IHC, and IHC-P. https://www.novusbio.com/products/tktl1-antibody_nbp1-31674
5. Anti-alpha-tubulin antibody: Application statement in manufacturer's website as following: this anti-alpha-tubulin antibody is validated for use in WB, IP, IHC, IF, FC, CoIP, and ELISA. https://www.ptglab.com/products/TUBA1B-Antibody-11224-1-AP.htm
6. Anti-beta-tubulin antibody: Application statement in manufacturer's website as following: this anti-beta-tubulin antibody is validated for use in WB, IP, IHC, IF, FC, CoIP, and ELISA. https://www.ptglab.com/products/TUBB-Antibody-10094-1-AP.htm
7. Anti-CD4 antibody: Application statement in manufacturer's website as following: this anti-CD4 antibody is validated for use in IHC. https://www.genetech.com.cn/goods/goods_detail/234.html
8. Anti-CD8 antibody: Application statement in manufacturer's website as following: this anti-CD8 antibody is validated for use in IHC. https://shop.leicabiosystems.com/en-cn/ihc-ish/ihc-primary-antibodies/pid-cd8
9. Anti-HER-2/NEU (4B5) antibody: Application statement in manufacturer's website as following: this anti-CD8 antibody is validated for use in IHC. https://pim-eservices.roche.com/eLD/api/downloads/aaa1f711-6207-eb11-0091-005056a71a5d?countryIsoCode=gb

Eukaryotic cell lines

Policy information about cell lines

Cell line source(s)

Human HEK293T (Cat# CRL-11268 from ATCC; RRID: CVCL_QW54), MKN45 (Cat# JCRB0254 from Japanese Collection of Research Bioresources (JCRB) Cell Bank, RRID: CVCL_0434), MGC803 (Cat# C6582 from Beyotime Biotechnology, RRID: CVCL_5334), and NCI-N87 (Cat# CRL-5822 from ATCC; RRID: CVCL_1603), were obtained.

Authentication

Cells validation using short tandem repeat markers (STR) were performed by Meixuan Biological Science and Technology Ltd. (Shanghai). In detail, these cell lines were firstly tested cell species by PCR method using extracted total genomic DNA, and examined by STR profiling. Then, STR data were analyzed using the DSMZ (German Collection of Microorganisms and Cell Cultures) online STR database (http://www.dsmz.de/fp/cgi-bin/str.html).

Mycoplasma contamination

Cell lines were tested negative for mycoplasma contamination.

Commonly misidentified lines
(See ICLAC register)

No commonly misidentified cell lines were used.

Human research participants

Policy information about studies involving human research participants

Population characteristics	The discovery cohort for proteome profiling consisted of 206 patients with GC, including the DOS subcohort (44 cases treated with S-1 and oxaliplatin combined with docetaxel), the XELOX subcohort (70 cases treated with capecitabine and oxaliplatin), and the HER2 subcohort (71 cases treated with the anti-HER2-based therapy), according to the treatment profiles. Another 21 cases were assigned as "Others," of which 3 cases received apatinib or docetaxel therapies, and 18 cases had no chemotherapy information. Tumor response was assessed and categorized as a complete response (CR), partial response (PR), stable disease (SD), or progressive disease (PD). Here, the ORR, defined as PR plus CR, was selected for the efficacy evaluation; patients with CR and PR were defined as sensitive (S) and those with SD and PD were defined as non-sensitive (NS). The discovery cohort contained S (n = 82) and NS (n = 103), males (n = 155) and females (n = 50), with a median age of 63, consistent with the distribution of gender and age for GC. For Lauren's type in the discovery cohort, 18.93% of GC patients were diffuse, 22.33% were mixed type, and 50% were intestinal. Median overall survival follow-up was 12 months (range, 0.7–77.6 months). 96.6% of GC patients were Grade II-III. Clinicopathological indicators, including age, gender, grade, Lauren's type, primary site, chemo-/targeted therapies, therapy cycle (3 weeks per cycle), RECIST, TNM stage, status of cancer recurrence or progression, and status of survival, are summarized in Supplementary Table 1 and Supplementary Data 1.
Recruitment	To obtain the proteomic characterization of the gastric cancer response to chemo- and targeted therapies, we performed strict screening process on gastric cancer patients based on the standard treatments including XELOX therapy, DOS therapy, and anti-HER2-based therapy. At first, we screened 2,943 gastric cancer patients with therapies or surgeries from January 2002 to Match 2018 in the Department of Pathology of Zhongshan Hospital, Fudan University (Shanghai, R. P. China). Then, we excluded 2,737 patients with postoperative chemotherapy, chemoradiotherapy, none complete pathological information, and other factors. All the chemotherapy regimens were given at standard dosing according to guidelines for gastric cancer patients on National Comprehensive Cancer Network (https://www.nccn.org/). Lastly, we assembled two cohorts, of which archival FFPE tissues with at least 80% tumor purity of GC cases were taken from chemotherapy naive patients. The discovery cohort for proteome profiling consisted of 206 patients with GC, including the DOS subcohort (44 cases treated with S-1 and oxaliplatin combined with docetaxel), the XELOX subcohort (70 cases treated with capecitabine and oxaliplatin), and the HER2 subcohort (71 cases treated with the anti-HER2-based therapy), according to the treatment profiles. Another 21 cases were assigned as "Others," of which 3 cases received apatinib or docetaxel therapies, and 18 cases had no chemotherapy information. The independent validation cohort for PRM verification included 60 patients with GC, receiving either DOS (N = 20), XELOX (N = 20), or anti-HER2 (N = 20) therapies. Written informed consent was received from all patients included in this study.
Ethics oversight	This study was approved by the Research Ethics Committee of Zhongshan Hospital (B2019-200R). Written informed consent was received from all patients included in this study.

Note that full information on the approval of the study protocol must also be provided in the manuscript.

Flow Cytometry

Plots

Confirm that:

- [x] The axis labels state the marker and fluorochrome used (e.g. CD4-FITC).
- [x] The axis scales are clearly visible. Include numbers along axes only for bottom left plot of group (a 'group' is an analysis of identical markers).
- [x] All plots are contour plots with outliers or pseudocolor plots.
- [x] A numerical value for number of cells or percentage (with statistics) is provided.

Methodology

Sample preparation	For cell cycle analysis, approximately 10^6 MKN45 cells were fixed in 4°C pre-cooled 70% ethanol overnight at 4°C. Following three washes, cells were incubated for 1h at 37°C in PBS with DNase-free RNase A (100mg/mL) and propidium iodide (50mg/mL). For cell apoptosis analysis, approximately 10^6 MKN45 cells were harvested from culture dishes using trypsin (without EDTA). After two washes with cold PBS, cells were resuspended in 100 μL 1 × Binding Buffer (Annexin V-FITC/PI Apoptosis Detection Kit, YEASEN, Shanghai, China). Cells were stained with 5 μL annexin V-FITC and 10 μL PI staining solution (Annexin V-FITC/PI Apoptosis Detection Kit) in the dark, at room temperature for 15 min. Following this incubation, 400 μL 1 × Binding Buffer was added to each sample, and then kept on ice until analysis (within 1 h).
Instrument	Beckman Coulter flow cytometer (Beckman Coulter, Brea, CA, USA).
Software	FlowJo version 10.7.1 (Becton Dickinson Life Sciences) .
Cell population abundance	Purity was determined to be >95% in all samples.
Gating strategy	Preliminary FSC/SSC gating was used to gate on the cell population.

- [x] Tick this box to confirm that a figure exemplifying the gating strategy is provided in the Supplementary Information.
